# The effects of an individualized exercise intervention on body composition in breast cancer patients undergoing treatment

**DOI:** 10.1590/S1516-31802007000100005

**Published:** 2007-01-04

**Authors:** Claudio Battaglini, Martim Bottaro, Carolyn Dennehy, Logan Rae, Edgar Shields, David Kirk, Anthony Hackney

**Keywords:** Exercise, Neoplasms, Adipose tissue, Skinfold thickness, Muscles, Exercício, Neoplasias, Tecido adiposo, Dobras cutâneas, Músculos

## Abstract

**CONTEXT AND OBJECTIVE:**

Changes in metabolism have been reported in the majority of patients undergoing cancer treatment, and these are usually characterized by progressive change in body composition. The effects of aerobic exercise programs to combat the cancer and cancer treatment-related side effects, which include the negative changes in body composition, have been extensively reported in the literature. However, few resistance exercise intervention studies have hypothesized that breast cancer patients might benefit from this type of exercise. The purpose of this study was to determine whether exercise protocols that emphasize resistance training would change body composition and strength in breast cancer patients undergoing treatment.

**DESIGN AND SETTING:**

Randomized controlled trial, at the Campus Recreation Center and Rocky Mountain Cancer Rehabilitation Institute of the University of Northern Colorado, and the North Colorado Medical Center.

**METHODS:**

Twenty inactive breast cancer patients were randomly assigned to a 21-week exercise group (n = 10) or a control group (n = 10). The exercise group trained at low to moderate intensity for 60 minutes on two days/week. The primary outcome measurements included body composition (skinfold method) and muscle strength (one repetition maximum).

**RESULTS:**

Significant differences in lean body mass, body fat and strength (p = 0.004, p = 0.004, p = 0.025, respectively) were observed between the groups at the end of the study.

**CONCLUSION:**

The results suggest that exercise emphasizing resistance training promotes positive changes in body composition and strength in breast cancer patients undergoing treatment.

## RESUMO

**CONTEXTO E OBJETIVO:**

Alterações no metabolismo têm sido apresentadas pela literatura e são comumente caracterizadas pela mudança progressiva da composição corporal observada na maioria de pacientes de câncer em tratamento. A literatura reporta os efeitos de programas de exercícios aeróbicos para combater o câncer e seus efeitos colaterais, porém, poucos estudos relacionados à intervenção por meio de exercícios de resistência em pacientes com câncer de mama e seus efeitos na composição corporal foram reportados na literatura. O objetivo foi determinar se um protocolo de exercícios de resistência traz alterações na composição corporal e força de pacientes com câncer de mama.

**TIPO DE ESTUDO E LOCAL:**

Estudo randomizado com um grupo de controle da University of Northern Colorado, Campus Recreation and Rocky Mountain Cancer Rehabilitation Institute e North Colorado Medical Center.

**MÉTODOS:**

20 pacientes portadoras de câncer de mama foram divididas aleatoriamente em um grupo que realizou 21 semanas de exercícios (n = 10) e um grupo de controle (n = 10). O grupo experimental realizou exercícios de intensidade baixa e moderada durante 60 minutos duas vezes por semana. A composição corporal foi avaliada pelo método de dobras cutâneas e a força msucular pelo teste de uma repetição máxima (1 RM).

**RESULTADOS:**

Foram encontradas diferenças significativas na massa magra, gordura corporal e força (p = 0,004, p = 0,004, p = 0,025, respectivamente) entre os grupos ao final do estudo.

**CONCLUSÃO:**

Os resultados deste estudo sugerem que exercícios com treinamento de força promovem mudanças na composição corporal e força em pacientes portadoras de câncer de mama sob tratamento médico.

## INTRODUCTION

Over 200,000 new cases of breast cancer are diagnosed each year in the United States, with roughly 40,000 of these cases resulting in the death of the patient.^1^ The most commonly used treatments for those with cancer are surgery, radiation, chemotherapy and hormone replacement therapies. However, all treatments for cancer cause various treatment-related side effects. Depending on the type and extent of the treatment needed, the symptoms can vary from acute to chronic. These side effects can include surgical complications, wound infection, loss of functional capacities, decreased range of motion, muscle weakness, osteoporosis, hot flushes, decreased immune system function, diarrhea, dyspnea, pain, numbness, nausea, overall fatigue, dry mouth, lung fibrosis, cardiomyopathies, vomiting, anorexia, hair loss, emotional/psychological disturbances and changes in metabolism. These can contribute towards marked changes in body composition.^2^

On the basis of observational studies, women with breast cancer who are overweight or gain weight after diagnosis are found to be at greater risk of breast cancer recurrence and death, compared with lighter women.^3^ This observation, first made in 1978, was surprising because of the typical nausea and vomiting associated with cancer treatment in this particular population.^4^ Although this finding was initially surprising, it has been reported consistently for more than two decades and has been attributed to many different physiological, behavioral and hormonal changes.^5,6^

In recent publications, the most frequently reported side effect observed with the administration of cancer treatment is fatigue.^7^ In fact, several studies have reported that fatigue affects approximately 70% of patients during chemotherapy and radio-therapy treatment.^8,9^ The debilitating fatigue experienced by cancer patients is believed to be caused by a constellation of side effects that are produced by the treatment and the cancer disease itself. It has also reported in the literature that this intense fatigue is one of the major contributors towards cancer patients’ decreased ability to perform simple activities of daily living and to participate in regular exercise programs, thereby resulting in significant changes to overall health and quality of life.^10^ Furthermore, some studies have reported that the sedentary lifestyle of cancer patients is highly associated with negative changes in body composition.^5,11,12^ Regardless of the mechanisms that are involved in the changes to the patient's body composition, weight gain associated with increases in body fat and loss of lean tissue may be hazardous and predispose the patient towards the development of chronic disease.

In cancer patients, the increase in body weight may represent an ever higher threat of developing chronic diseases, in comparison with healthy individuals, due to the fact that the patient's immunological and metabolic responses are already functioning at non-normal levels. Finally, although inconclusive, weight gain may adversely affect the risk of cancer recurrence.^3,6^ Therefore, it is very important to address the issue of changes in body composition in cancer patients, so as to assist patients in preventing the development of future chronic conditions that could potentially affect possible tumor relapse and survival.^13^

Exercise has been shown to be an important component of healthy lifestyles.^14^ Among innumerous health benefits, exercise has been shown to be a powerful tool in assisting regular exercisers to change their body composition. It is plausible that participation in regular exercise programs may also represent a possible avenue for improving the body composition of cancer patients, thus enhancing their overall health and, most importantly, their quality of life. As a result, numerous studies involving the administration of exercise for cancer patients have utilized aerobically-based exercise programs.^15-17^ However, aerobic-based exercise interventions do not specifically address the issue of loss of muscle mass and the reduction in overall body strength typically observed in cancer patients.

In general, for healthy individuals, resistance training programs enhance metabolism, improve muscle endurance and coordination, increase muscle strength, and promote positive changes in body composition, including the development of lean tissue.^18^ Few studies have incorporated a combination of resistance and aerobic training in exercise protocol designs for cancer patients.^19-23^ Given that cancer patients experience metabolic, strength and body composition changes,^24^ it may be of value to further explore the effects of exercise protocols that emphasize resistance training, in an attempt to maximize the benefits of exercise for combating these side effects.

## OBJECTIVE

The main purpose of this study was to assess the effects of an individualized exercise intervention emphasizing resistance training, on changes in body composition and muscle strength in breast cancer patients during treatment.

## METHODS

### Experimental design and subjects

This study used a randomized two-group design (exercise and non-exercise), with multiple measurements of the dependent variable (body composition) and data from two fitness assessments. The fitness assessments included a cardiovascular endurance variable, oxygen uptake (VO_2_), and a muscle strength variable, predicted one-repetition maximum (1-RM) from a submaximal protocol. These were both administered prior to surgery and the start of cancer treatment and again at the end of the experiment. Fat mass (FM) and lean body mass (LBM) were assessed by the skinfold technique.

The volunteers for this study consisted of 20 females divided into a control group (n = 10) and an exercise group (n = 10). These volunteers had recently been diagnosed with breast cancer, and designated for surgery and chemotherapy treatment. All the subjects were recruited from the northern Colorado region through oncology practices between January 2001 and April 2003. Patients were screened for participation based upon a physician's review of the patient's medical history and a physical examination. The criteria for non-participation in the study included the presence of cardiovascular disease; acute or chronic respiratory disease; acute or chronic bone, joint or muscle abnormalities (unless these diseases would not compromise the patient's ability to participate in the exercise rehabilitation program); metastatic disease; and immune deficiency.

The verbal and written explanations of the protocol included timelines outlining events during the investigation and the time when and location where each event would occur. Information regarding pre-assessment guidelines, risks and the subject's rights were given to each patient during the meeting with a member of the research team. The research team member asked each potential participant if they understood the information and if they were interested in participating in the study. Upon agreement, participants were then required to sign an informed consent form approved by the University of Northern Colorado's Internal Review Board (UNCO IRB) and the North Colorado Medical Center's Internal Review Board (NCMC IRB) outlining the purpose, procedures, benefits, risks and voluntary nature of this investigation, prior to their participation in the study. All subjects recruited after April 13, 2003, were asked to sign an authorization form under the Health Insurance Portability and Accountability Act of 1996 (HIPA) for the use or disclosure of protected health information for research.

### Assessment protocols

During the week after diagnosis and before surgery, the subjects were randomly assigned to two different groups. The first group was an experimental group and the second group was a control group. The randomization procedure involved the drawing of numbers by the patients, which ranged from 1 to 20. Subjects who drew even numbers were placed into the experimental group while subjects who drew odd numbers were placed into the control group. All subjects were assessed for body composition following surgery, throughout the study, and at the end of the experiment in week 21 (Table 1).

**Table 1.** Clinical assessment timeline for all breast cancer patients studied

Week	Exercise group
1	Biopsy and diagnosis meeting
	Introduction to the study and informed consent signing
	Pre-surgery assessment and randomization to groups
	Fatigue assessment; fitness assessment
2	Surgery
3	Recovery from surgery
4	Post-surgery assessment
	Fatigue assessment
5	
6	Exercise intervention begins
7	
8 (first dose of chemotherapy)	First assessment of fatigue during treatment
9	
10	
11	Second assessment of fatigue during treatment
12	
13	
14	
15	Third assessment of fatigue during treatment
16	
17	
18	
19	
20	
21	Final assessment
	Fatigue assessment; fitness assessmen

Skinfold measurements were used to assess changes in body composition during the study. The three-site skinfold measurement formula for women (triceps, suprailiac and abdomen) proposed by Jackson et al.^25^ was used for determining body composition. A Lange skinfold caliper (Cambridge Scientific Industries, Inc., Cambridge, Maryland) was the instrument used for assessing body composition. To minimize measurement error, all subjects were given pre-assessment guidelines and all skinfold measurements were performed only by the primary investigator. During the cancer treatment period, body composition assessments were performed at the oncologists’ offices approximately 30 minutes before administering the treatment, for both the exercise and the non-exercise groups. Lean body mass (LBM) was calculated using the following formula: LBM = body weight (BW) – fat mass (FM). The result from the LBM calculation was converted into relative lean body mass values (%LBM).

A fitness assessment comprising cardiovascular endurance and muscle strength tests was performed prior to surgery and at the end of the study, at the Rocky Mountain Cancer Rehabilitation Institute of the University of Northern Colorado, in Greeley, Colorado. Each patient wore an A3 Polar heart rate monitor (Lake Success, New York) to determine resting heart rate and to monitor heart rate responses during cardiovascular assessments, as well as for controlling intensities during exercise sessions. Height and body weight were assessed using a Detecto Model 437 Physician Beam Scale (Webb City, Missouri). Blood pressure was assessed using an ADC 922 series aneroid sphygmomanometer (Hauppauge, New York) and a Littmann Stethoscope (St. Paul, Minnesota). Cardiovascular endurance assessments were performed on a Quinton model 65 treadmill (Bothell, Washington).

The muscle strength assessments involved the utilization of the following exercises: leg extension, seated leg curl, lateral pulldown and seated chest press. The strength assessments were performed on LifeFitness (Schiller Park, Illinois) and Quantum (Stafford, Texas) weight training machines and also using free weights. Muscle strength assessments were performed using a submaximal muscle endurance protocol that predicts 1-RM and which was developed for middle-aged and older women.^26^ Each of the two fitness assessments was standardized and followed exactly the same sequence of events and protocols.

In an attempt to minimize any possible disappointment among subjects in the control group, a research team member explained to all subjects prior to the randomization process that, even though research has shown that exercise may benefit cancer patients undergoing treatment, there was still the need for more research to confirm or refute previous research findings. Also, all subjects assigned to the control group were given the opportunity, at the end of the study protocol, to engage in a supervised exercise program.

The control group was reminded to abstain from participating in any regular and/or supervised exercise program while participating in the study. During the body composition assessments, the primary investigator had the opportunity to reinforce the non-participation of the control group in regular exercise and to verify the adherence to the study protocol among the subjects in the control group.

### Exercise intervention protocol

The exercise intervention assigned to the experimental group started during week six of the experiment (after the recovery time following the surgery) and lasted until week 21 of the study (Table 1). Due to post-surgery cancer treatment schedules, all subjects assigned to the exercise group started their exercise routine approximately three weeks prior to the administration of their first cancer treatment. All the exercise sessions were conducted at the Rocky Mountain Cancer Rehabilitation Institute and/or at the University of Northern Colorado Campus Recreation Center. Subjects assigned to the experimental group exercised two times per week for a period of not more than 60 minutes. The rest period between sessions was at least 48 hours and no longer than 84 hours. The subjects followed an individually prescribed exercise intervention program that was designed in accordance with the exercise guidelines from the American College of Sports Medicine (ACSM) for special and elderly populations and the specific guidelines published in Exercise and Cancer Recovery.^14,27^

Because of the age range criteria for participation in the study, as well as the lack of specific guidelines for exercise among cancer patients, the above guidelines were believed to be the most appropriate ones for the population used in this investigation. All the subjects assigned to the exercise group performed exercises at sub-maximal intensities that were determined according to the results from their fitness assessments. These subjects performed exercises with intensities varying between 40% and 60% of their predicted maximum exercise capacity for both the cardiovascular and strength exercises. Each of the individualized exercise prescriptions was based on the results from the fitness assessment administered at the beginning of the study. All subjects were led and monitored during each exercise session by a trained cancer exercise specialist from the University of Northern Colorado School of Sport and Exercise Science who had participated in an educational seminar prior to the study.

The design of the exercise intervention included both cardiovascular and resistance training, as well as flexibility training. The format for each exercise session consisted of initial administration of a cardiovascular activity (approximately 6-12 minutes) that included walking on a treadmill and the use of a cycle ergometer or elliptical equipment, followed by whole-body stretching sessions (5-10 minutes), resistance training (15-30 minutes), and a cool-down period that included stretching activities for approximately 8 minutes. This format followed the ACSM guidelines for the components that should be included in a training session with the goal of promoting changes in body composition.^14^

The administration of resistance training was emphasized in the design because resistance training is the type of exercise that promotes changes in body composition and attenuates the loss of lean body mass that is usually associated with a variety of catabolic conditions, including cancer.^11^ For the resistance exercise portion of the exercise protocol, the intensities of the exercises were determined according to the results obtained in the first fitness assessment. During this part of the exercise session, eight to twelve different types of resistance exercises emphasizing all the major muscle groups were utilized. All the resistance exercises were performed using weight training machines, free weights (hand dumbbells), elastic bands, and/or therapeutic balls. The resistance exercises that were assigned to the exercise group included: lateral and frontal raises, horizontal chest press, late ral pulldown, alternating biceps curls with dumbbells, triceps extension, leg press, leg extension, leg curl, standing calf raises and three different types of abdominal exercises (forward crunches, oblique crunches, and lower abdominal crunches).

To develop a training effect, the increases in load during the experiment followed the ACSM progression models for resistance exercise training methods.^18^ The number of repetitions for each exercise ranged from six to twelve. The subjects performed a maximum of three sets of each exercise per session. During the first week, all subjects assigned to the exercise group performed only one set of each exercise prescribed for the sessions. During the exercise intervention, the subjects in the exercise group progressively advanced to perform two to three sets for each exercise, which then continued to be administered until the end of the experiment. The movements for each exercise were performed at a moderate speed (three seconds for the concentric phase and three seconds for the eccentric phase of the movement during each repetition for each exercise). The rest interval period between each set and between each exercise varied from 30 seconds to 1 minute, or according to the subject's needs.

### Statistical analyses

Significant changes in the exercise and control groups regarding body composition and overall muscle strength between the time of diagnosis and the end of the study were analyzed using two-way mixed model analysis of variance (ANOVA; between versus within). The dependent variables included in the ANOVA design were %LBM, %FM and overall muscle strength. The latter was defined as the sum of the results from the predicted 1-RM obtained from assessing the exercises of leg extension, seated leg curl, lateral pull down and seated chest press, prior to surgery and again at the end of the experiment. The probability level for statistical significance was set at p < 0.05 for all comparisons. The data were entered into a personal computer and the statistical procedures were performed using the Statistical Package for the Social Sciences (SPSS) version 10.0. Descriptive statistics were expressed as means ± standard deviations (SD).

## RESULTS

All the participants in the control group (n = 10; age 56.6 ± 16.0 years; body mass 82.2 ± 25.0 kg; height 169.2 ± 10.2 cm; FM 30.1 ± 4.2 %), and in the exercise group (n = 10; age 57.5 ± 23.0 years; body mass 77.5 ± 27.3 kg; height 168.9 ± 10.2 cm; FM 20.0 ± 3.4 %) completed the study protocol. Therefore, the adherence rate among all the subjects was 100%. No cases of injury or any cancer treatment complications impeded subjects in the exercise group from completing the exercise protocol two times a week. Only one subject in the exercise group missed one week of exercise, and this was due to readjustment of her prosthetics implanted following cancer surgery. However, this patient resumed her participation in the study the following week, and completed the protocol with no other complications.

No significant differences in %LBM in either group over time was observed from the assessments administered following surgery, during treatment and at the end of the experiment: F-value, F (1, 18) = 3.394 with p = 0.82 (α = 0.05). However, it was found that there was a significant interaction effect between the groups and %LBM over time (p = 0.000).

The results from the *post hoc* analyses revealed significant differences in %LBM between groups at the end of the experiment (p = 0.004). The results from the *post hoc* analyses are presented in Table 2.

**Table 2.** Difference in relative lean body mass (%LBM) between groups of breast cancer-treated patients

Time of measurement	Control group (n = 10)	Exercise group (n = 10)	Mean difference (%)	p-value
First measurement: post-surgery	69.1 ± 4.2	71.0 ± 3.4	- 1.1	0.53
Second measurement: treatment starts	70.0 ± 4.0	72.0 ± 3.1	- 1.2	0.23
Third measurement	69.4 ± 4.4	72.5 ± 2.9	- 3.1	0.07
Fourth measurement	69.6 ± 3.8	72.8 ± 3.1	- 3.2	0.05
Final measurement	68.9 ± 4.1	74.1 ± 2.9	- 5.2	0.004

Even though there was no significant changes in % LBM observed in either group over time (p = 0.82), the exercise group did demonstrate a slim but clinically sound increase in %LBM, in comparison with the values obtained during the first measurement, while the control group experienced a slight reduction in %LBM (Table 2).

No significant differences in relative body fat (%BF) were observed in either group from the assessments administered following surgery, during treatment and at the end of the experiment: F-value, F (1, 18) = 3.353 with p = 0.84 (α = 0.05). However, there was a significant interaction effect between the groups and %BF over time (p = 0.000). Significant differences between the groups were observed at the final measurement (p = 0.004). The results from the *post hoc* analyses are displayed in Table 3.

**Table 3.** Difference in relative body fat (% BF) between groups of breast cancer-treated patients

Time of measurement	Control group (n = 10)	Exercise group (n = 10)	Mean difference (%)	p-value
First measurement: post-surgery	30.1 ± 4.2	29.0 ± 3.4	1.1	0.53
Second measurement: treatment starts	29.9 ± 4.0	28.0 ± 3.1	1.9	0.23
Third measurement	30.3 ± 3.9	27.4 ± 2.9	2.9	0.07
Fourth measurement	30.4 ± 3.8	27.3 ± 3.0	3.1	0.05
Final measurement	31.2 ± 4.1	25.9 ± 2.9	5.3	0.004

Even though no statistical significance was found in either group regarding changes in %BF over the course of the study, the exercise group did experience a 10.89% decrease in %BF during the experiment, while the control group experienced a 3.52% gain in %BF.

The exercise and control groups were both assessed for the variable of overall muscle strength prior to surgery and at the conclusion of the exercise intervention at the end of the experiment. The variable of overall muscle strength in this experiment was defined as the sum of the results from the predicted 1-RM that was obtained from assessing the exercises of leg extension, seated leg curl, lateral pulldown and seated chest press, prior to surgery and again at the end of the experiment. The descriptive statistics for the overall muscle strength of each group are presented in Table 4.

**Table 4.** Descriptive data for muscle strength assessments during treatment (mean ± standard deviation, SD) of breast cancer-treated patients

Time of measurement	Control group (n = 10)	Exercise group (n = 10)
First measurement (Pre-surgery)	262.49 ± 40.87	269.77 ± 12.77
Final measurement	260.89 ± 38.76	295.59 ± 22.65

There were no significant changes in overall muscle strength in either group over time: F-value, F (1, 18) = 2.340 with p = 0.144 (α = 0.05). However, there was a significant interaction effect between the groups and overall muscle strength over time (p = 0.000). Significant differences in overall muscle strength between the groups were observed at the final assessment measurement at the end of the study (p = 0.025). Even though there were no significant changes within the groups from the first measurement to the last, the exercise group did demonstrate a 9.57% increase in overall strength, in comparison with the values obtained at the first measurement, while the control group experienced a reduction of 0.61% in overall strength.

## DISCUSSION

According to the American Cancer Society, an estimated 212,000 new cases of breast cancer were expected to be found among women in the United States during 2005.^1^ This is an alarming statistic, but the actual death rate among women with breast cancer has decreased over recent years because of early detection and more advanced treatment options.^1^ Even though many forms of therapy have been developed to treat breast cancer, numerous side effects develop during cancer treatments.^1,5^ While some of these side effects are not preventable, researchers have explored ways of combating many of the side effects, including fatigue, strength reductions, and negative changes in lean body mass.^15,17,19,21,22^ Evidence from these studies supports exercise as a method for reducing the negative changes in body composition and strength that are observed in the majority of patients undergoing treatment. However, few studies have examined the impact of resistance training on changes in body composition and strength in breast cancer patients. Therefore, the purpose of the present study was to observe the effects of an individualized prescriptive exercise program emphasizing resistance training, on changes in body composition and muscle strength in breast cancer patients during treatment.

The present study investigated whether the exercise and control groups experienced changes in body composition during the period between the post-surgery assessment and the completion of the research protocol. Additionally, this study examined whether there were significant differences in body composition between the exercise and control groups during the study. The results from the analyses showed that there were no significant changes in body composition (%LBM and %BF, p = 0.82, p = 0.84, respectively) over time in either the exercise or the control group. However, the changes in %LBM and %BF were revealed to be significantly different between the groups at the final assessment administered at the end of the study (p = 0.004, p = 0.004 respectively). At the end of the study, the exercise group was seen to have experienced a 10.89% decrease in the percentage of body fat and an increase of 4.26% in the percentage of lean body mass, while the control group had a 3.52% gain in percentage of body fat and a slight reduction in percentage of lean body mass of 1.42%. These results are in agreement with other studies that have reported that exercise intervention programs improve body composition in cancer patients undergoing treatment.^17,24,28,29^

In the study by Kolden et al.,^28^ 40 breast cancer patients participated in a 16-week exercise intervention that was very similar to that of the present experiment. These authors also found that weight and percentage of body fat were not significantly improved at the end of the intervention period and ascribed their non-significant results to the small sample size. In the present study too, the small sample size may be the reason for the non-significant differences observed in both groups regarding changes in body composition. However, a possible trend involving the gains in %LBM and reductions in %BF that were experienced by the exercise group, and the opposite results observed in the control group during the study, is very promising. The percentage changes in body composition presented by this study are of clinical importance. Even though no statistically significant differences were found in either group, an increase of 10.89% in lean body mass and a reduction of 4.26% in body fat in the exercise group may have great clinical implications for the success of cancer treatments, as well as for patients’ overall quality of life.

In a study with similar exercise duration but a larger sample size, Schwartz^29^ followed 78 women with breast cancer. This author examined the effects of aerobic exercise on weight gain during the first four cycles of chemotherapy treatment. Overall, the women who adhered to the exercise program maintained their pre-cancer weight, while those who did not participate in the exercise program steadily gained weight during the study.^29^ Unfortunately, detailed descriptions of the intensity or frequency of the exercise program implemented in the study were not included. Additionally, body composition measurements were not taken to determine whether the weight changes in that study were due to changes in percentage of body fat and/or lean body mass.

In a study by Winningham et al.,^17^ 24 breast cancer patients undergoing treatment were randomly assigned to either a group with an organized aerobic exercise routine or to a control group where exercise was not administered. The results from the patients in the exercise group were compared to those in the control group over a 10-12 week period. Regarding the type of aerobic exercise protocol, the subjects completed 20-30 minutes of moderately high aerobic exercises (60-85% of age-predicted maximum) on three days of each week. These authors reported that the exercise group in the study experienced increases in lean body mass, while the control group did not. Additionally, they found a 0.5% decrease in body fat in the exercise group. These small differences in body composition may be explained by the higher intensity used by those authors during that study, which differed from the intensities used in the present study.

Segal et al.^24^ utilized a larger sample size, including 123 breast cancer patients in a structured exercise training protocol. In their study design, the subjects in the exercise group (roughly half of all the subjects) did aerobic training five times a week for 26 weeks, which was longer training consisting of more intense exercises than in previous studies that explored exercise in cancer populations, and also in relation to the present study. After finding significant improvements in physical functioning and reduced body weight in the exercise group (p = 0.01 and p < 0.05, respectively), these authors concluded that breast cancer patients can tolerate a more intense exercise regimen and that increases in physical activity can significantly assist breast cancer patients in body weight management during treatment.

According to the results from the study by Segal et al.,^24^ the intensity utilized in the present study could have also contributed to the non-significant differences found in body composition during the 15.5 weeks of the exercise protocol. Therefore, it is recommended that the present study should be reproduced to include a larger sample size, with a longer and more intensive exercise protocol, so that a more conclusive explanation can be given regarding the benefits of an exercise program emphasizing resistance training, on changes in body composition.

A secondary purpose of the present study was to examine whether there were significant differences in muscle strength between the exercise and the control groups at the end of the experiment. No significant differences in overall muscle strength were observed in either group during the study (p = 0.144). However, significant differences in muscle strength were observed between the groups at the end of the study (p = 0.025). Even though no statistically significant results were observed in either group between pre-surgery and the end of the study, there was a percentage increase in overall strength in the exercise group over the course of the intervention, while the control group experienced a slight reduction in overall muscle strength.

Previous studies have shown an overall trend of positive associations between exercise and strength changes in cancer patients. Adamsen et al.^19^ examined the effects of a six-week, high-intensity, combined aerobic and resistance exercise protocol on 23 cancer patients. Even though this protocol was of short duration, the subjects experienced gains in muscle strength over the course of the study, with a mean gain of 33%. However, these findings might be explained by the intensity of the activities performed by the cancer patients (cycling at 60-100% of age-predicted maximum and three sets of five to eight repetitions at 85-95% 1-RM).

Segal et al.^30^ carried out the largest study utilizing a resistance-training protocol. One hundred and fifty-five cancer patients performed an exercise routine consisting of resistance training alone (two sets of 12 repetitions at 60-70% 1-RM), three days a week for 12 weeks. The subjects demonstrated a mean increase of 42% in upper body strength and 36% in lower body strength.^30^

In another study, Durak and Lilly^21^ used a 10-week, mixed-mode exercise protocol with an unspecified intensity, to examine whether it was possible for 20 cancer patients to experience strength gains while undergoing treatment. These authors also completed another mixed exercise study, but with 25 cancer patients for a total of 20 weeks.^22^ Surprisingly, there were no significant differences in the results in either of the two studies by Durak et al.,^21,22^ but there were improvements in strength in each of the exercise groups (43% and 45%, respectively). The findings from these authors’ two studies differ from those of the present study. Given that the exercise protocols utilized were similar to what was used in the present study (in which the patients exercised two times per week), the explanation for the difference in results between the studies may be attributed to the moderate intensity utilized by Durak et al.^21,22^ In the present experiment, the patients did not achieve moderate exercise intensities until approximately three weeks before the end of the study. The lower intensities adopted over most of the duration of the present study varied between 40% and 50% of the predicted maximal capacity and therefore may have slowed down the development of muscle strength in the exercise group. Although no significant changes in muscle strength over time were observed in the present study, the significant differences between the groups that were observed at the end of the study confirm the efficacy of the exercise training protocol for maintaining and possibly developing muscle strength in breast cancer patients undergoing treatment.

Breast cancer patients undergoing treatment have been shown to experience negative body composition and strength changes. While many of the causes for these negative side effects are still a topic of great debate among researchers, some intervention methods have been presented in the recent literature. The results from studies involving the administration of exercise as an intervention for cancer patients suggest that positive changes in body composition and muscle strength can be achieved during treatment.

## CONCLUSION

The results suggest that exercise emphasizing resistance training promotes positive changes in body composition and strength in breast cancer patients undergoing treatment. During the present study, the changes in body composition and muscle strength showed a positive trend toward statistical significance. As the cancer treatment progressed, the changes and differences between the groups became more evident. The indication of a possible trend between the effects of an exercise protocol emphasizing resistance training, on changes in body composition and muscle strength in breast cancer patients undergoing treatment, suggests that this study should be reproduced. Certain considerations such as a larger sample size, a different strength assessment protocol, the utilization of higher exercise intensity, and a longer-duration exercise protocol should be explored.
