# Erratum

**DOI:** 10.1590/S1516-31802007000500014

**Published:** 2007-09-02

In the article "The effects of an individualized exercise intervention on body composition in breast cancer patients undergoing treatment", by Battaglini C, Bottaro M, Dennehy C, Rae L, Shields E, Kirk D, Hackney A [Sao Paulo Med J. 2007;125(1):22-8], we wish to communicate that the correct name of the last author should be Anthony Carl Hackney.

Therefore, the correct way to cite a reference to this article is: Battaglini C, Bottaro M, Dennehy C, Rae L, Shields E, Kirk D, Hackney AC. The effects of an individualized exercise intervention on body composition in breast cancer patients undergoing treatment. Sao Paulo Med J. 2007;125(1):22-8.

